# Flying foxes create extensive seed shadows and enhance germination success of pioneer plant species in deforested Madagascan landscapes

**DOI:** 10.1371/journal.pone.0184023

**Published:** 2017-09-06

**Authors:** Ryszard Oleksy, Luca Giuggioli, Thomas J. McKetterick, Paul A. Racey, Gareth Jones

**Affiliations:** 1 School of Life Sciences, University of Bristol, Bristol, United Kingdom; 2 Department of Engineering Mathematics, University of Bristol, Bristol, United Kingdom; 3 Centre for Ecology and Conservation, College of Life and Environmental Sciences, University of Exeter, Cornwall campus, Penryn, Cornwall, United Kingdom; Università degli Studi di Napoli Federico II, ITALY

## Abstract

Seed dispersal plays a significant role in forest regeneration and maintenance. Flying foxes are often posited as effective long-distance seed dispersers due to their large home ranges and ability to disperse seeds when flying. We evaluate the importance of the Madagascan flying fox *Pteropus rufus* in the maintenance and regeneration of forests in one of the world’s priority conservation areas. We tested germination success of over 20,000 seeds from the figs *Ficus polita*, *F*. *grevei* and *F*. *lutea* extracted from bat faeces and ripe fruits under progressively more natural conditions, ranging from petri-dishes to outdoor environments. Seeds from all fig species showed increased germination success after passing through the bats’ digestive tracts. Outside, germination success in *F*. *polita* was highest in faecal seeds grown under semi-shaded conditions, and seeds that passed through bats showed increased seedling establishment success. We used data from feeding trials and GPS tracking to construct seed shadow maps to visualize seed dispersal patterns. The models use Gaussian probability density functions to predict the likelihood of defecation events occurring after feeding. In captivity, bats had short gut retention times (often < 30 mins), but were sometimes able to retain seeds for over 24h. In the wild, bats travelled 3–5 km within 24–280 min after feeding, when defecation of ingested seeds is very likely. They produced extensive seed shadows (11 bats potentially dispersing seeds over 58,000 ha over 45 total days of tracking) when feeding on figs within their large foraging areas and dispersed the seeds in habitats that were often partially shaded and hence would facilitate germination up to 20 km from the feeding tree. Because figs are important pioneer species, *P*. *rufus* is an important dispersal vector that makes a vital contribution to the regeneration and maintenance of highly fragmented forest patches in Madagascar.

## Introduction

Tropical deforestation is a major cause of global environmental change [1], is becoming more rapid [2] and can reduce biodiversity substantially [3]. The maintenance and regeneration of tropical forests is a key conservation issue, and identifying natural mechanisms that promote forest regeneration is important. Long-distance dispersal events are important for the dispersal of seeds of forest species in the landscape, yet quantifying long-distance seed dispersal can be challenging [4–9]. Combining telemetry data with gut retention times for seeds can be valuable in calculating seed dispersal distances [10–13]. The conservation of seed dispersers at population sizes that maintain their ecological function to promote seed dispersal at large spatial scales has been identified as an important research topic in changing landscapes [14]. Frugivorous bats have recently been identified as being important for the dispersal of pioneer tree and shrub species from forests to grassland in Neotropical landscapes [15], and a recent GPS study highlighted the potential importance of pteropodid bats as long-distance dispersers in Africa [12]. Here we estimate seed dispersal distances using GPS data at high spatial and temporal resolution, and evaluate the importance of flying foxes for dispersing pioneer forest species over large spatial scales in one of the world’s biodiversity hotspots, Madagascar.

Madagascar is unique in terms of its rich biodiversity and high endemism. Isolated for over 80 million years from the mainland, it possesses an array of extraordinary species adapted to a wide variety of habitats [16]. Humans have been present for at least 4000 years [17] and initiated extinction of the megafauna and deforestation [e.g. 16, 18–20]. Currently, 70% of the entire island is covered by homogenous grassland [20] and by 2025 the remnant forest fragments situated outside protected areas may be almost completely lost [21].

Rapid deforestation in Madagascar has had profound effects on its wildlife. The highlands are almost entirely devoid of native animals, which mostly depend on forested habitats [20]. Forest fragmentation is extremely high with 45% of remaining forest comprised of patches <500 km^2^ in area [22]. Increasing fragmentation of Madagascar’s forested habitats decreases the diversity of frugivores that are unable to survive in isolated forest patches. With a lack of animal-mediated seed dispersal, the patches gradually change their composition towards more generalist, wind-dispersed species and become botanically impoverished [23]. The lack of large herbivores, the limited seed dispersal abilities of arboreal primates and the vulnerability of the avifauna to forest degradation, all contribute to a low capacity for the regeneration of Madagascar’s forests.

Here we aimed to evaluate whether the endemic flying fox *Pteropus rufus* can potentially promote forest regeneration in Madagascar. We compared the germination success of bat-processed seeds and the establishment of seedlings of pioneer fig species *Ficus polita*, *F*. *grevei* and *F*. *lutea* extracted from bat faeces and rejecta (spat-out) pellets with those of ripe fruits (unprocessed seeds) in progressively more natural challenges. *P*. *rufus* ingests the small seeds found in figs after crushing the fruit against the palate, consuming the fluids and soft parts of the fruit, then spitting the fibrous fruit coating out. The seeds are then dispersed when the bats defaecate. These fig species rapidly colonise degraded areas, and can grow into relatively large trees, or exist as stranglers. Information from gut retention times (GRTs) estimated after seed ingestion experiments is integrated with information from fine-scale movements of 11 individual *P*. *rufus* bats tracked over 45 days while carrying high resolution GPS tags. We used Gaussian probability density functions to predict the likelihood of defecation events occurring after feeding, creating seed shadow maps that predict the probability of seed deposition from previously known feeding locations. Our approaches allow us to test the hypothesis that through increased germination of bat-ingested seeds and their dispersal over extensive areas flying foxes such as *P*. *rufus* can promote forest regeneration in areas threatened by deforestation such as Madagascar.

## Materials and methods

### Germination experiments

We aimed to determine if fig seeds germinated at a higher rate after passing through the digestive systems of *P*. *rufus*. The germination experiments were conducted in Mandena Conservation Zone (24° 57' 0" S, 46° 59' 0" E) between September and October 2011 for *F*. *polita*; in Berenty Private Reserve (25° 00’ 33” S; 46° 18’ 29” E) between August and September 2012 for *F*. *grevei* and in Marovitsika village (18° 50’ 27” S; 48° 3’ 18” E) between February and April 2014 for *F*. *lutea*. The first two sites are in southwest Madagascar, and Marovitsika is in the northeast of the country. Berenty and Marovitsika experience equatorial climates, while Mandena has a monsoon climate. Mandena is part of Madagascar’s national network of protected areas, and encompasses around 148 ha of fragmented and degraded littoral forest. Berenty consists of 200 ha of gallery forest along the Mandrare River. Much of the area is arid and dominated by spiny forest. It is surrounded by ca. 30,000 ha of commercial sisal plantations. At Marovitsika work was conducted in a private eucalyptus plantation with remnants of primary forest fragments in which bats roost (usually in valleys). The eucalyptus is at different stages of growth.

*F*. *polita* seeds were collected between 1–30 August 2011, *F*. *grevei* seeds between 23 July-17 August 2012, and *F*. *lutea* seeds in February and March 2014. Three times per week visits were conducted at roosts and feeding sites where droppings were produced by a large number of bats (for example the Berenty roost contained over 600 bats when studied). All experiments were performed in the dry season.

Seeds were extracted from bat faeces and rejecta pellets collected from under the roost (one roost per site) and at feeding sites of *P*. *rufus*. All the seeds manually extracted from bat faeces in Mandena were identified as *Ficus polita*; in Berenty as *F*. *grevei* and in Marovitsika as *F*. *lutea*. Additionally, seeds were extracted from fresh fruits to act as a control (unprocessed seeds). Seeds from fresh fruit were only discarded if they were damaged by fig wasps with such damage resulting in the loss of a gelatinous seed coat, which normally facilitates ingestion by the bats.

We tested if differences in germination success occurred in seeds that had passed through bat guts (and were extracted from faeces) compared with seeds from fresh fruit. In Petri dishes (91 mm) seeds (faecal, rejecta and unprocessed) were sown on filter paper (100 mm) with 20 seeds in each treatment replicated 15 times. Dishes were moistened with 3 ml of water and then when necessary, with all dishes treated consistently. For *F*. *polita* each Petri dish was sealed with clear Vaseline to prevent desiccation. Since some seeds became infected by fungi when sealing dishes with Vaseline, this approach was abandoned in experiments on the other two fig species and dishes were checked every second day, while once weekly watering was performed if necessary. Petri dish experiments were conducted at each site independently, and dishes were placed on outside tables each shaded by a fabric roof canopy. In total 20,700 seeds were tested. Seeds were collected from at least three trees/fig species, with at least 50 fruits collected per tree.

We tested whether germination success differed in seeds from bat faeces, seeds from rejecta pellets, and seeds from fresh fruit in three different shade regimes. In the shade control and outside environment experiments (performed at Mandena in September 2011), *F*. *polita* seeds (faecal, rejecta and unprocessed) were sown on unsterilized littoral soil in plastic open-topped bags (10 x 15 cm; 20 seeds in each of 15 replicates/treatment) and placed in direct sun, semi-shade on the edge of the forest and shade inside a thick forest. Because of time constraints, we were only able to perform this experiment with *F*. *polita*. A Generalized Linear Model was used in PASW Statistics 18 with number of germinating seeds as the dependent variable, a Poisson probability distribution (because many bags showed zero germination) and a log-link function. Including an interaction term between the shade and seed type treatments gave a lower Akaike’s Information Criterion (AIC) value (and hence better fit) than a model that did not include the interaction term, and so the interaction was included in the model.

In July 2012, 10 months after *F*. *polita* seeds were sown, we revisited the site where bags with the seedlings were left outside to test whether seeds that had passed through bat guts showed higher rates of establishment.

### Gut retention time (GRT)

Between August and September 2012, nine bats were kept for observations in cages (ca. 1 x 1 x 1 m) for up to three nights. The bats were captured at feeding sites using handmade nylon mist nets (5m x 2m: 10 cm mesh) and transported up to 3 km away to the Berenty Private Reserve in cotton bags. Each bat was kept separately and water was provided *ad libitum* during the whole period of the study. During the first night the bat usually refused to eat, therefore the food was provided on the second night at 18.00h when measurements commenced. We assumed that the gut was empty at that time. Four bats settled in the cages, and accepted food so could provide data for estimates of GRT. Each bat was given 200 g of sliced banana. Each slice was either covered with seeds extracted from ripe *F*. *grevei* fruits (one fig per slice, containing 200–300 seeds) or left plain as a control. The slices given to each bat were equal in number and size and were provided once per night at 18.00h and leftover food removed at 06.00h. The cages with bats were checked every 30 min starting from 18.30h and the number of slices eaten and droppings produced were recorded and removed. The droppings were checked for presence of seeds and their number recorded. We arbitrarily considered 25 seeds as a threshold for a high seed density in faeces.

### Dispersal distances

The frequency of dropping production obtained from GRT experiments was used for calculations of seed dispersal distances based on GPS movements of bats tracked in Berenty Private Reserve. The GPS devices (E-Obs; Germany; ca. 26 g) were glued on bats between the shoulders after trimming the underlying fur to about 0.5 cm length. Non-irritant skin bonding glue (Ostomy Adhesive Solution, Salts Healthcare, UK) was used and applied directly to the skin of the bats. The tags recorded the GPS position of bats every 2.5 min when bats were moving (the tags contain acceleration sensors to document when the bats are stationary or in flight) and every 30 min when they were resting. The median spatial accuracy of the fixes was 14.4 +/- 4.39 (SD) m (n = 3 tags, 370 fixes) [24]. Details of the study area, dates of data collection and characteristics of the study area are provided in [24]. Data on foraging times and travel distances are taken from the complete sample of 15 bats described in that paper (four adult males, five adult females, five immature males and one immature female). For seed shadow calculations, we used data from 11 bats with more than four nights of movement data (three adult males, five adult females and three immature males), and excluded four bats that did not feed on figs and restricted their foraging to the sisal plantation. The total number of tracking nights in the seed shadow study was 45 (only nights when bats fed on figs were included). All bats studied weighed at least 600g, and so tag masses were always less than 5% of body mass.

### Seed shadows

The GPS tracks of bats viewed in Google Earth (Google Inc. 2013) showed feeding trees used by bats. Ground truthing showed that all sites where bats rested during the tracking sessions (16 sites) contained *Ficus* species (sometimes >1 tree/site), and *Ficus* seeds were the only seeds found in large quantities in droppings at the time of the study. To calculate seed dispersal probability and seed shadow maps, spatial location and temporal (time of fix) data from GPS-recorded trajectories (bat movement tracks) of 11 individual bats over 45 nights of tracking were used. GPS tracks were combined with the probability distributions for defaecation events following seed ingestion. By assuming diffusive displacement (details in Supporting Information), it was possible to estimate where an individual might have been as a function of time. gut retention times (GRTs) estimated after seed ingestion experiments. By associating the probability of defaecation events along the animal tracks it was possible to calculate the spatial area around previously visited feeding locations where seeds could be found, with 90% probability after the first defaecation events and 30% probability after the second, third or fourth defaecation events (see Supporting Information). We also determined in which habitats seeds were most likely to be deposited. For this, habitat definitions are provided in [24].

## Results

### Germination success

**Fig 1:**
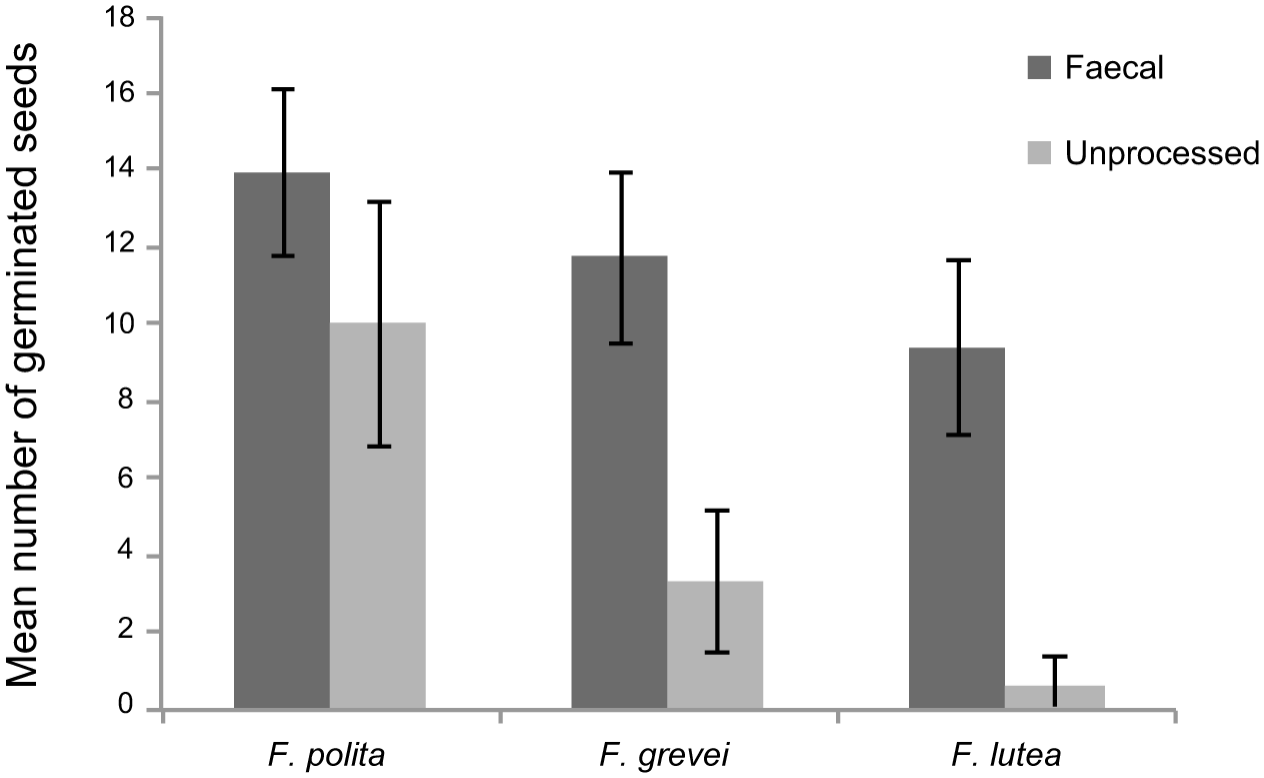
Germination success of fig seeds on filter paper. Mean germination success for *Ficus polita*, *F*. *grevei and F*. *lutea* for faecal (dark shading) and for unprocessed seeds removed from ripe fruits (light shading) sown on filter paper. There were 15 replicates of 20 seeds/ treatment. The error bars are standard deviations.

**Fig 2:**
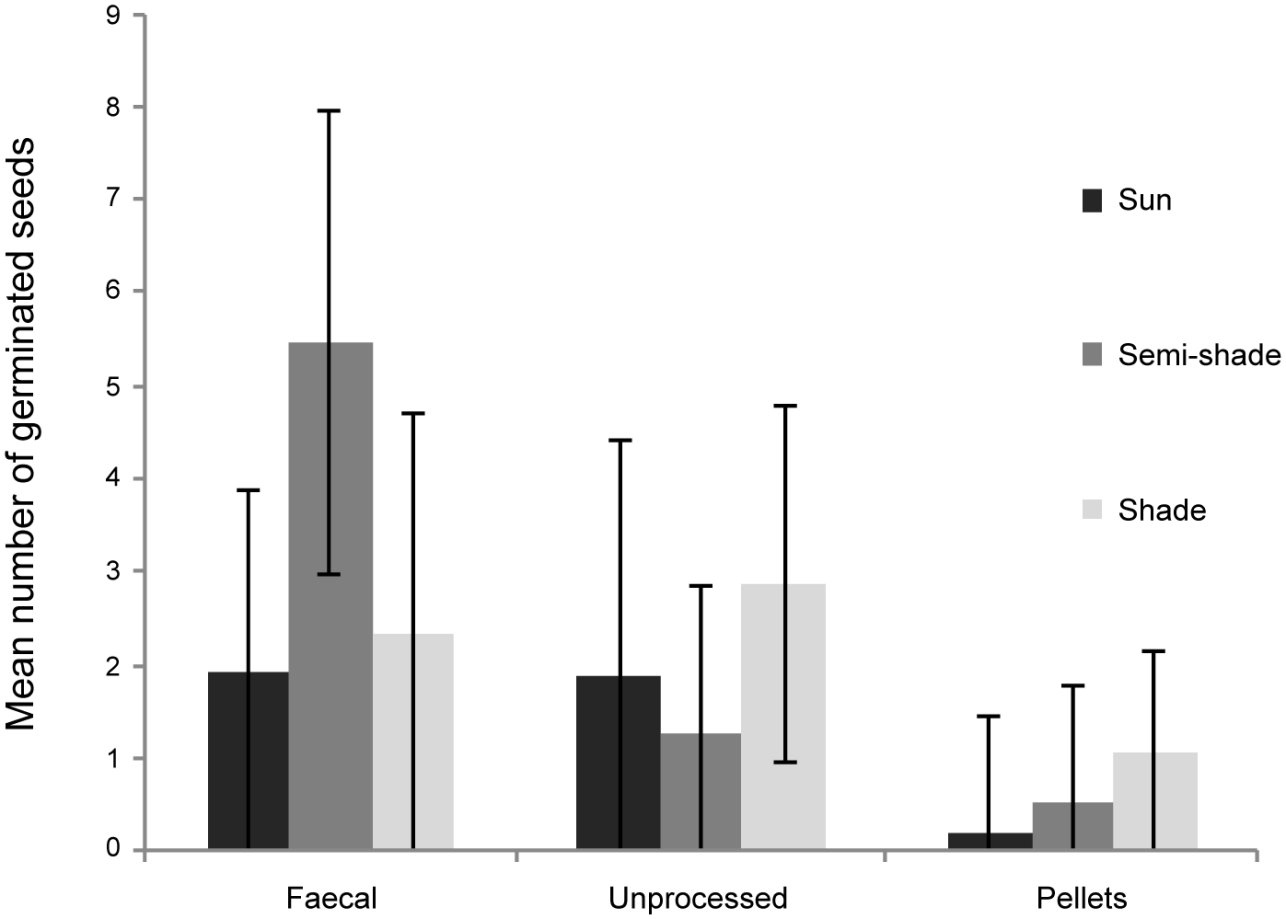
Germination success of fig seeds in semi-natural conditions. Mean germination success of *Ficus polita* faecal, unprocessed and from rejecta pellet seeds under three different light intensities. Each treatment was replicated 15 times with 20 seeds/replicate (2,700 seeds tested). The bars represent standard deviations.

In all three fig species (Fig 1) bat-processed seeds germinated on filter paper with higher success rates than unprocessed seeds (data for mean numbers of seeds germinating in 15 replicates of 20 seeds for each species: *F*. *polita t*_28_ = 3.96, *P* < 0.001; *F*. *grevei t*_28_ = 11.1, *P* < 0.001; *F*. *lutea t*_28_ = 14.3, *P* < 0.001). Seeds from rejecta (spat out pellets) failed to germinate in Petri dishes. An experiment on seed germination under different shade conditions in an outside environment was performed only on *F*. *polita* and germination success was significantly affected by the seed type (processed vs. unprocessed—Wald Chi-square statistic = 50.65, 2 df, *P* <0.001), by shade treatment (Wald Chi-square statistic = 10.22, 2 df, *P* = 0.006) and by the interaction between seed type and shade (Wald Chi-square statistic = 113.98, 2 df, *P* <0.001). Germination success was highest in faecal seeds grown under semi-shaded conditions (mean 5.47 ± 2.47 SD), and this treatment resulted in higher germination success compared with all other treatments (pairwise comparisons *P* <0.001 based on Wald Confidence Intervals) (Fig 2). Interestingly, semi-shaded conditions increased germination success only for faecal seeds (as shown through a significant interaction between seed type and shade). The lowest germination success was for seeds recovered from rejecta pellets.

### Sapling establishment

Only seedlings growing in semi-shaded condition survived and out of all 900 seeds (45 bags) planted in this treatment, 28 saplings established. Of these, 24 saplings established from 11 bags came from faecal seeds (8% of 300 planted seeds or 73.3% of 15 bags) and only four saplings established in two bags came from unprocessed seeds (1.33% out of 300 seeds or 13.3% of 15 bags). The rate of establishment for bat-processed seeds was higher than expected if processed and unprocessed seeds had equal rates of establishment (*X*^2^_1_ with Yates’ correction = 12.9, *P* < 0.001).

### Gut retention time

**Fig 3:**
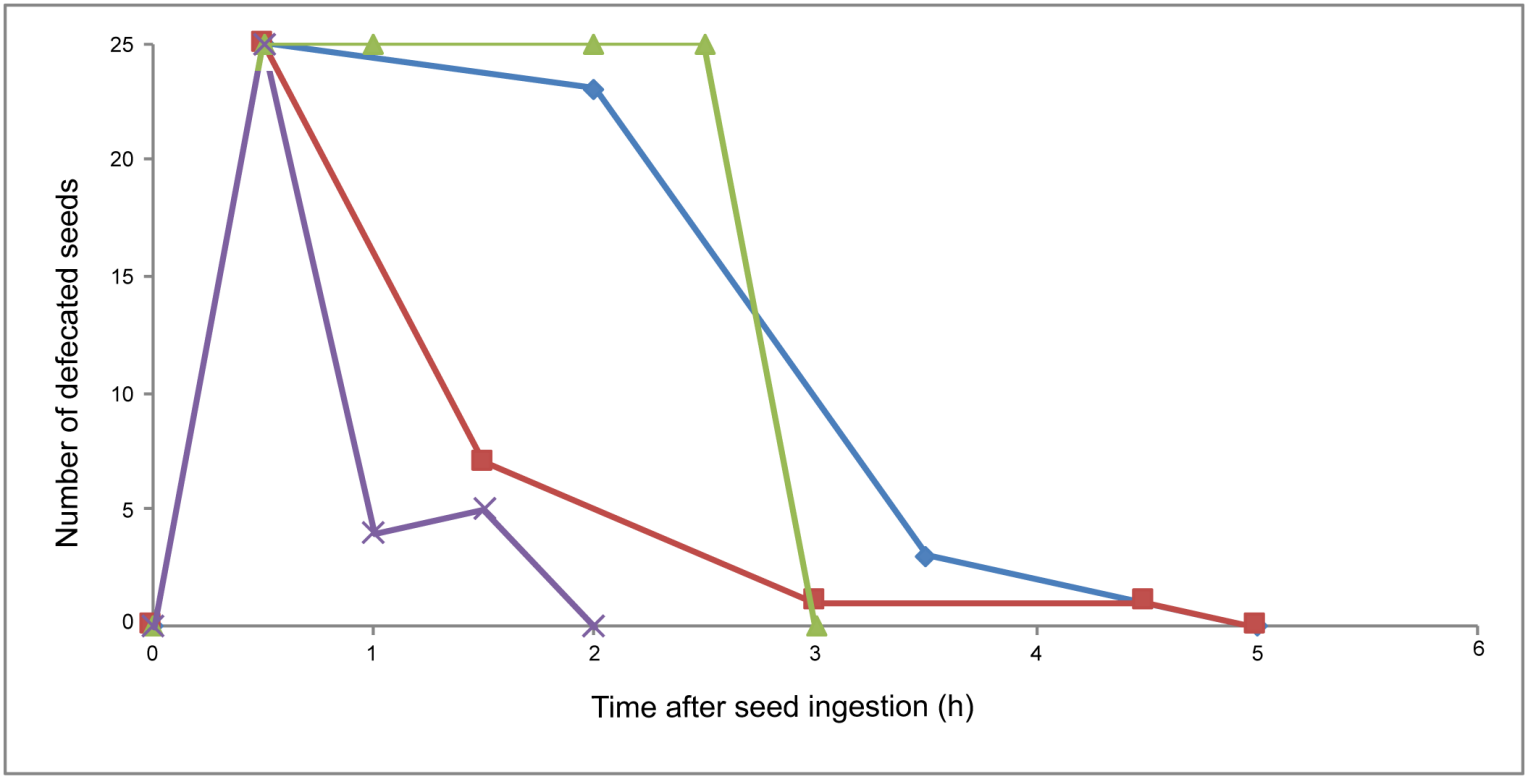
Gut retention times of Madagascan flying foxes. Seed retention time in four colour coded individual bats. The vertical axis represents number of fig seeds present in the faeces, the maximum of 25 refers to a score of ‘25 and over’. The horizontal axis represents time since the last feeding of a bat (seed ingestion). Measurements were made every 30 mins for up to 6 h after food was presented (after this time droppings were produced rarely). The bats produced 63 droppings in total.

There was considerable variation among individuals in GRTs (Fig 3). On average most faeces with more than 25 seeds were produced within 30 mins of feeding (Fig 3). We thus used 30 mins as the GRT in our seed shadow models. An average of 2.5 droppings were produced in the 30 min sample sessions, so GRTs may be less than 30 mins, and as short as 12 mins if droppings were produced at even intervals over the 30 min period. Some seeds were also retained in the bat’s gut for over 20 h (n = 5 occasions), with the longest recorded period of 24.5 h for a defaecation containing 13 seeds. On average, faeces containing at least 25 seeds can remain in the gut for 6.6 h (± 8.04 SD, n = 5) and smaller numbers of seeds can be retained for over 15 h (4 seeds ± 6.3 SD; 15.21 h ± 6.28; n = 5).

### Travel distances

**Fig 4:**
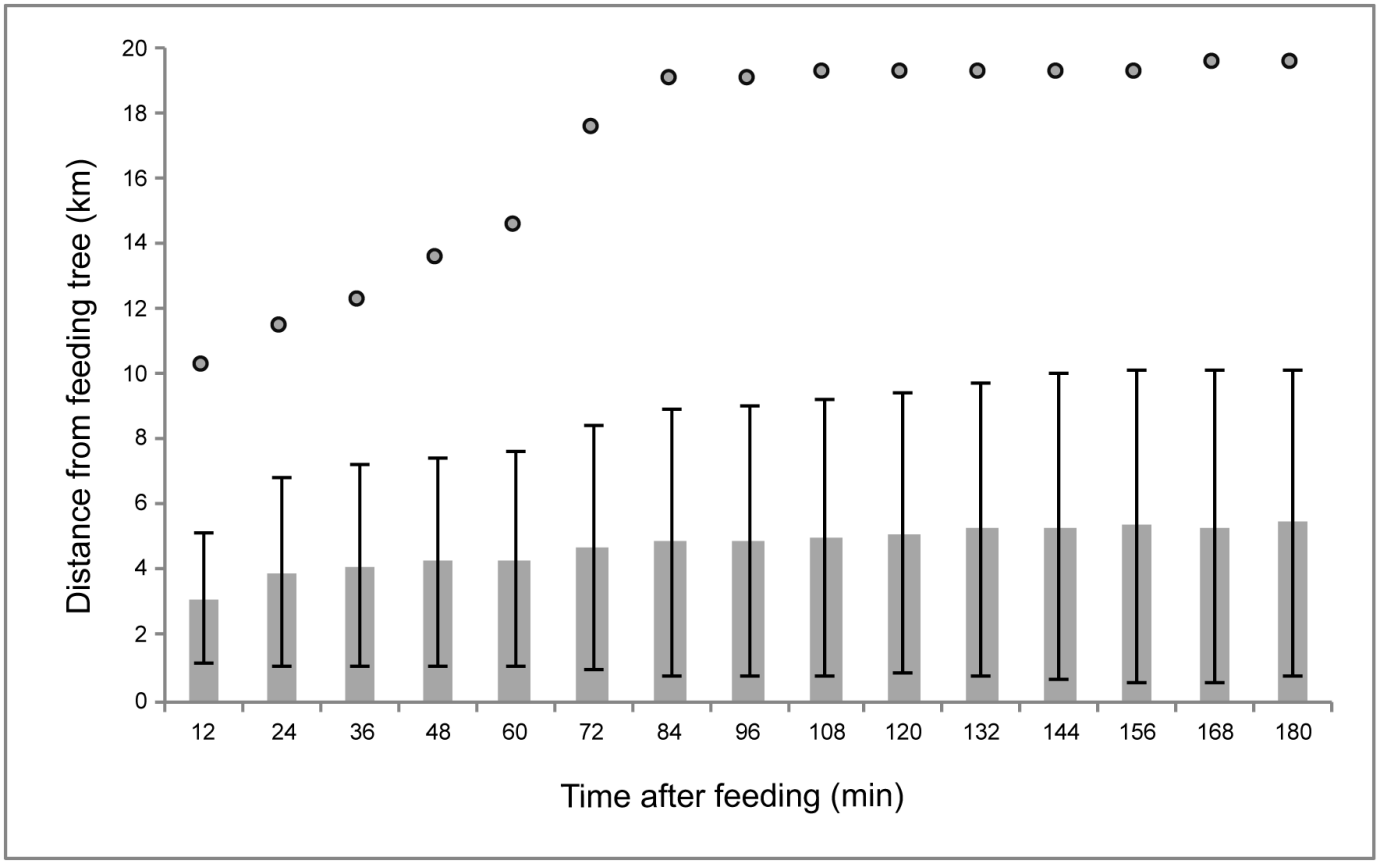
Travel distances of Madagascan flying foxes. Distances travelled by 11 *Pteropus rufus* bats from fig trees for up to 3 h after feeding (the time in which most droppings containing more than 25 seeds were produced in our GRT study–see Fig 3). The position of the bat was considered every 12 min as a straight line along its flight path in relation to the feeding tree it used. The bars represent average recorded distance from the feeding sites (n = 71) with the standard deviation. The circles indicate the maximum distance recorded.

Bats travelled up to 10 km from the food source within only 12 min (the assumed minimum GRT) (Fig 4). Travel distance increased rapidly at first and reached an asymptote within 36 min of feeding. The maximum distance bats travelled from a feeding tree was between 19 and 20 km and this distance was covered within 84 min on five occasions. Overall, bats travelled 3–5 km within 24–280 min after feeding. The distance between the roost and feeding sites was very variable. There were *Ficus* trees next to the roost in which bats would first feed, but they could travel as far as 20 km to another feeding site in the same night. On average bats travelled 5.4 (±4.29 SD) km within 3 h after feeding when the defecation of ingested seeds is the most probable based on the GRT measurements recorded. Bats foraged for over nine hours per night (9.44 h ± 1.49 SD, n = 15), typically with over two hours of commuting flight to and from the feeding sites (2.54 h ± 0.9 SD, n = 15). In other words 26.9% of a bat’s time budget was allocated to flight between feeding sites, during which it can disperse seeds away from the parental trees.

### Seed shadows

**Fig 5:**
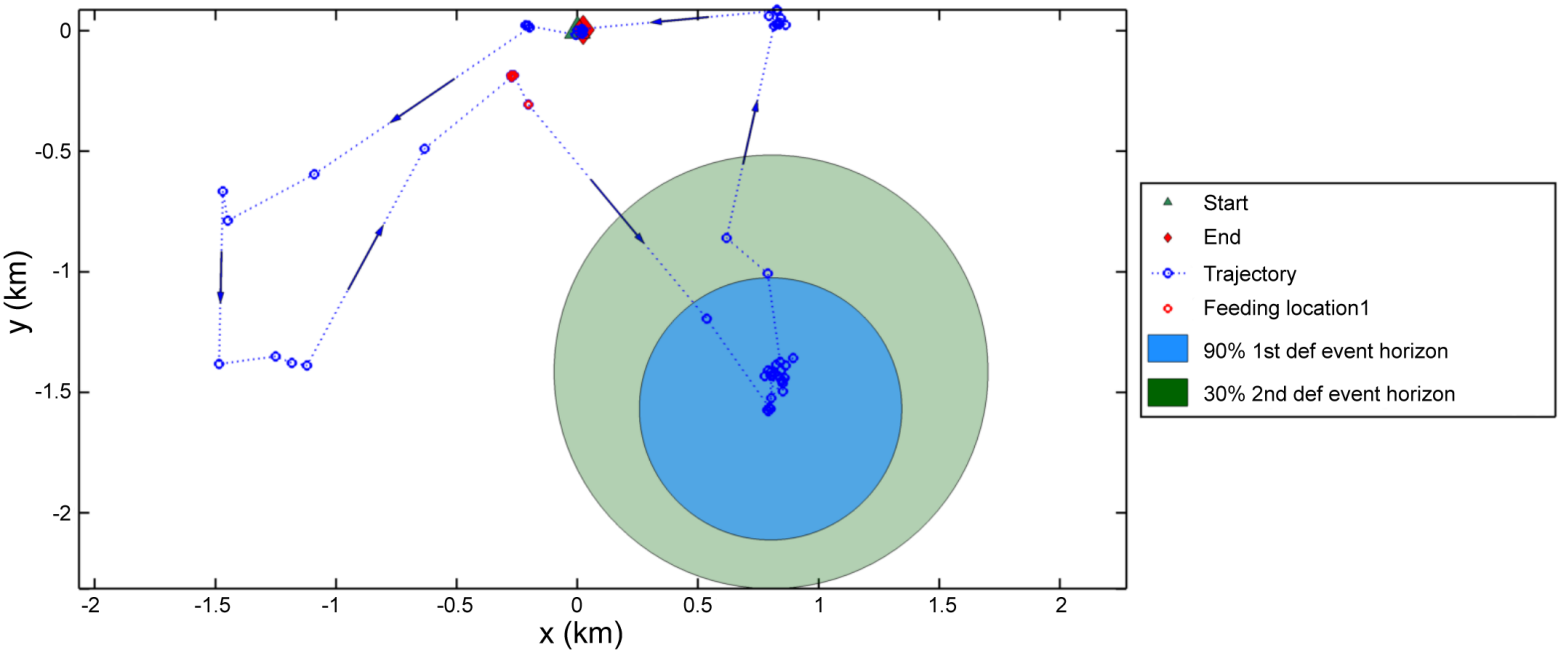
Seed shadow plot from one feeding event. Sample trajectory with one single feeding event (12 hours: 18.00h-06.00h). The spatio-temporal data (GPS fixes in blue) have been plotted at the same resolutions as recorded. The start and end points of the trajectory have been marked (the roost at 0,0 km) as well as the first and subsequent defecation event horizons. Notice that the two circles are not concentric. Concentric circles would appear only if the individual would have returned at time *T*_2_ at the same location where it was estimated to be at time *T*_1_.

**Fig 6:**
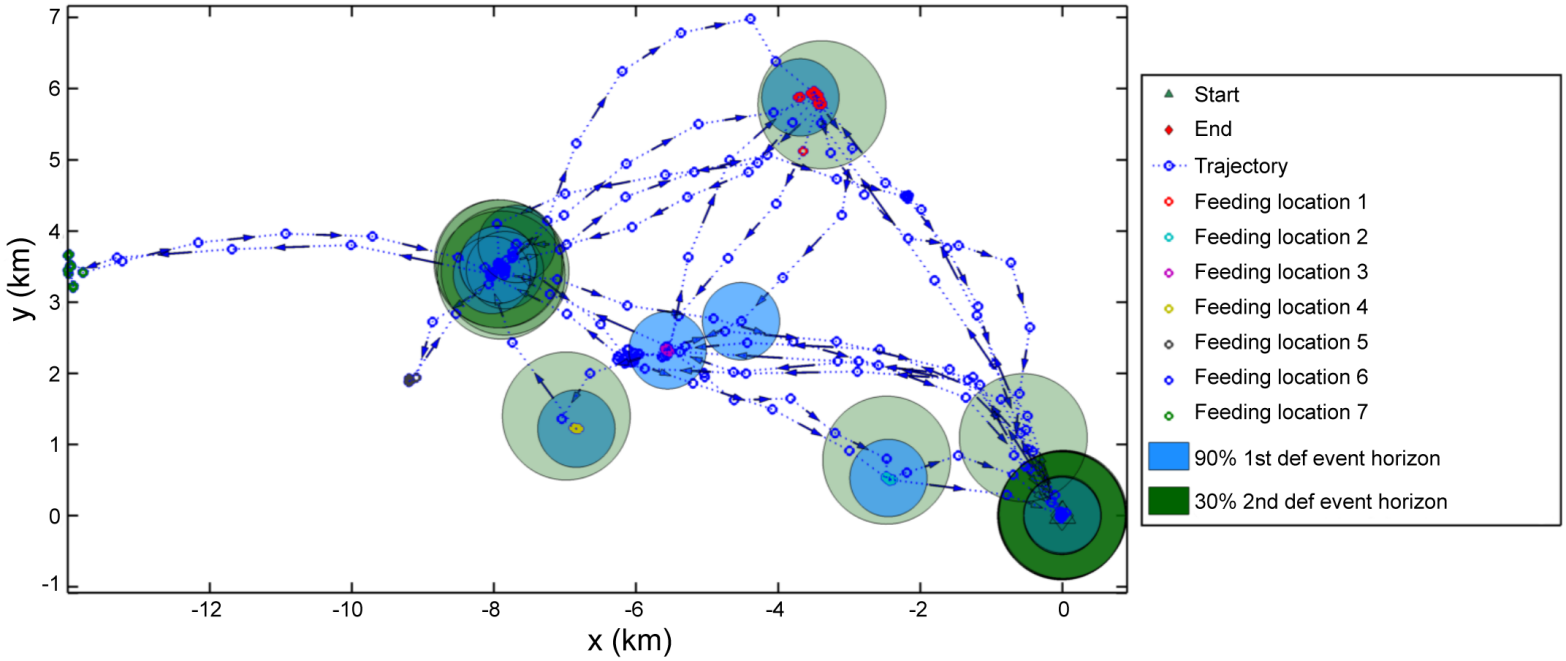
Seed shadow plot with multiple feeding events. Sample trajectory with several different feeding events and locations from one bat covering nearly a week of feeding. The start and end of the trajectory are at the roost (0,0) with two defecation event horizons shown for each feeding event. The light green and blue colour indicates a single defecation event while the darker colours indicate more than one overlapping circle due to several defecation events. The small blue dots are GPS fixes.

**Fig 7:**
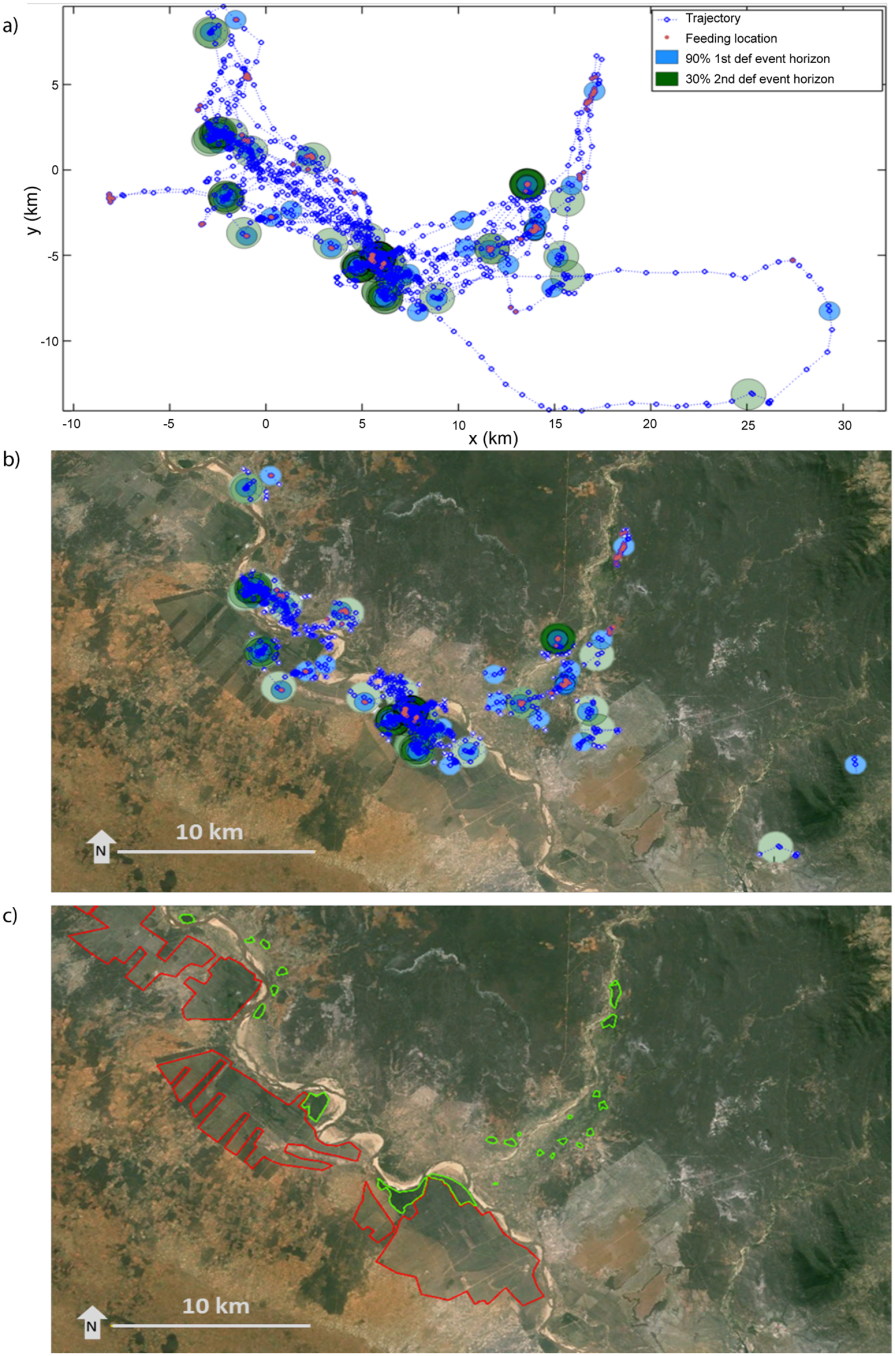
Seed shadow plots from all bats combined. The combined data of all 11 bats (45 nights of feeding) are plotted relative to the average position (a) and overlaid on a Google Earth map of the area (b) with major sisal plantation (red) and forest fragments (green) indicated (c). The rest of the habitat is composed of agricultural land, clearings, spiny forest or small groups of trees. All feeding locations are marked and the two defecation event horizons plotted corresponding to each feeding event. Light green and blue indicates a single defecation event (30% and 90% defecation probability respectively) while the darker colours indicate accumulations of several defaecation events. In (b) and (c) map data: Google, DigitalGlobe.

After estimating the probability of defaecation events, seed shadows were constructed for each recorded feeding event within a trajectory (bat movement track). One shadow corresponded to the area with a 90% probability of defecation and the other with a 30% probability of any second, third or fourth subsequent defecation (see Figs 5 and 6 for sample trajectories and Fig 7 for the combined results of trajectories from all 11 bats). The seed shadow plot is based around an assumption that within 30 min there is a 90% probability that a bat will defecate after feeding. Hence the diffusive model (S1 File) shows that the likelihood of defaecation varies from around 10 min to almost an hour with a peak probability at 30 min (as this is the time by which we are certain bats produce droppings). This temporal variability in the likelihood of dropping production forms the boundaries of the 90% and 30% defaecation event horizons in Figs 4–7.

The seed shadow map (Fig 7) indicates that 11 bats can disperse seeds covering an area of over 58,000 ha within 45 days. However, seed dispersal is concentrated in an area covering around 22,500 ha. High accumulations of seeds occur in the central part of the area, which is close to the roost and around relatively few feeding trees (as indicated by the dark green and dark blue colours in Fig 7). Many seeds are dispersed away from the parental trees with 90% probability of defaecation (as indicated by the blue circles). The seeds are dispersed in a mosaic of habitats. From 17 major areas with 90% probability of the first defaecation event, five are located solely within sisal plantations, three are in sisal with gallery forest fragments, seven are in gallery forest fragments while two are in spiny forest. Many (at least 70%) of these situations are shaded or semi-shaded.

## Discussion

We report several novel findings that are important for seed dispersal in degraded habitats in the Old World tropics. First, we show that the germination success of fig seeds (which are important pioneer species), and the establishment of their seedlings, increases as a consequence of the seeds passing through the guts of flying foxes. Second, by using a combination of GRT and movement ecology data, we show that Madagascan flying foxes are important long distance seed dispersers, and frequently disperse seeds in degraded habitats, potentially facilitating forest regeneration. Below we discuss these aspects further.

Among all experiments the best germination results were achieved using filter paper where the Petri dishes provided a stable (albeit unnatural) environment. Seeds of *F*. *polita* extracted from the spat-out pellets (rejecta) had the lowest success. In most cases no, or very low, germination rates were evident. The spat-out seeds are empty husks and are mostly those already killed by fig wasp larvae (R.Z. Oleksy pers. obs.). As a result they lose their gelatinous coating, which makes it difficult for the bats to swallow the seeds, so they are rejected along with the fruit fibre, in the form of rejecta (spat-out) pellets [25]. Picot et al. (2007) confirmed the absence of germination from rejecta seeds [26]. The rejection of non-viable seeds may also enhance the success of *P*. *rufus* as a disperser of fig seeds compared with animals that do not reject non-viable seeds. Because we discarded seeds that showed evidence of fig wasp damage from ripe fruit, the higher germination success of seeds from faeces compared with seeds from ripe fruit is unlikely to be explained by the ripe fruit containing more non-viable seeds. In *F*. *polita*, faecal seeds sown in semi-shaded conditions achieved significantly greater germination success than those in direct sun or completely shaded conditions. It is expected that apart from faecal material remaining on the seeds, shade helps retain moisture in soil and this is necessary for seeds to grow. Although the number of germinated seeds was rather low in different shade treatments, the approach took into account such factors as predation and atmospheric changes, as the seeds were sown outside with no protection. Therefore, the seedlings were exposed to heavy rain and fluctuating humidity, as well as to many invertebrates that feed on seeds and seedlings. Given that at least 70% of seed shadows fell within or close to forested areas (i.e. often in semi-shaded conditions), this will further increase the likelihood of seed germination.

A higher germination success for bat-processed seeds has been suggested previously [26] where *Ficus* seeds processed by the Madagascan fruit bat *Eidolon dupreanum* germinated better than unprocessed seeds (germination rates of 20% for *F*. *brachyclada* and 40% for *F*. *pyrifolia* while no seeds germinated from ripe fruits). The germination of *F*. *lutea* and *F*. *natalensis* seeds was higher after ingestion by *P*. *voeltzkowi* compared with germination rates of intact seeds [27]. However, both studies involved relatively small sample sizes and their germination experiments were minor additions to detailed studies of the bats’ diet. Seeds that pass through the digestive tract of animals may lose pericarp and mucilaginous coats that serve as substrates for fungal and bacterial infection, and may soften the seed coat after mastication or after exposure to acids and enzymes in the digestive tract [28, 29]. Additionally, animals remove the fruit pulp from the seeds, which usually contains germination inhibitors [30]. Faecal material that adheres to seeds may also provide nutrients that enhance germination success.

Our experiments showed that bat-processed *F*. *polita* seeds germinated better in a relatively natural environment than unprocessed seeds and were more successful in terms of relatively long-term survival. Because *Ficus* species are often described as ‘keystone’ species in tropical ecosystems [e.g. 31], they have been promoted as framework species for tropical forest restoration [32]. *Ficus* produce fruit all year round, usually when other fruits are scarce [33]. They attract a wide range of frugivores, enabling them to survive during times of food shortage [e.g. 34]. Many *Ficus* species are fire-resilient and drought- and pest-resistant, and survive under the harsh conditions found in many degraded areas [35]. Additionally, their root systems can often penetrate even the hardest substrates (including rocks) improving its aeration and drainage, and making it suitable for the establishment of other plant species [36]. These characteristics of *Ficus* species, combined with the large number of fig seeds deposited by bats in a wide range of habitats increase the potential for germination and establishment, yet the propagation and use of figs as a tool to restore forest has been little studied [37]. Fruiting fig trees will in turn attract more frugivores, which contribute to the soil seed bank and thus facilitate passive restoration. Given its ability to disperse fig seeds and increase their germination success, *P*. *rufus* may act as a keystone species in regeneration of the forest in Madagascar. Our study focused on small seeds of *Ficus* species: larger seeds may be deposited directly under feeding trees after the fruit flesh is ingested. Qualitative analyses of droppings suggested that *P*.*rufus* were eating solely *Ficus* fruits at the time of study, although it is possible that the bats fed on some larger fruits and ate only the pulp, which could not be detected in faeces.

Because on average 2.5 droppings were produced in the first 30 min sample, average food transit time in *P*. *rufus* may be as short as 12 min. If all droppings were produced near the end of the inspection intervals, transit times may be closer to 30 min, which is the probability peak of the first defaecation event we have modelled here. Either way, it is clear that the transit time is short. This corresponds with previous findings in which food transit time in *Pteropus*, *Ptenochirus*, *Nyctimene*, *Lissonycteris*, *Epomops* and *Rousettus* species was recorded to be between 12 and 114 min (and is usually less than 30 min) [e.g. 25, 38–43]. A recent study on *Eidolon helvum* indicated a gut passage time between 4 to 1143 min with median of 72 min [12]. Our probability density function models incorporated a range of GRTs with a peak probability of first defaecation at 30 mins.

Several studies have, like ours, estimated gut transit times using extracted seeds inserted into slices of other fruits such as papaya or banana [33,41] or using fluorescence dye on intact fruits [12]. As bananas are not commonly eaten by *P*.*rufus* the species and ripeness of fruit might affect gut passage times, especially due to the content of secondary plant compounds (or presence of dye), which may speed up or slow down gut passage [44,45]. The seeds with longer retention times may be dispersed over greater distances and in a larger variety of habitats than those with short GRTs [46]. We acknowledge that there are biases inherent in studies of GRTs, and such biases will affect our seed shadow estimates. However, such biases are inherent in most studies performed to date. Gut retention times will influence the capability of animals to disperse seeds and are likely to be species-specific and related to anatomy and physiology [46]. Our study tested only one seed species on a limited number of individuals. The food provided for the bats (bananas) is not commonly eaten by the species and may influence GRT. Ideally it would have been informative to measure retention times of fig seeds from intact ripe fig fruits. However, the bats refused to eat these in captivity. By placing the fig seeds on banana slices we were able to control the numbers of seeds given to the bats, but the texture of banana could differ substantially from that of fig potentially altering the time over which fig seeds pass through the bat guts. Additionally, captivity (and any associated stress) may alter the bats’ behaviour.

Despite short defecation intervals in *P*. *rufus*, the bats can retain some seeds in the gut for over 20 h. Long retention times were also recorded for *Cynopterus sphinx*, where seeds were retained for more than 18 h [43]. Observations made on *Rousettus* captured around dusk showed that they defecate dark, viscous faeces that sporadically contained seeds. The faeces lacked the characteristic colour and texture of the eaten fruits, suggesting that they were retained in the gut during the day [44]. Also *P*. *poliocephalus* defecated when captured at dusk, suggesting long GRTs [43]. The feeding trials on *P*. *rufus* revealed similar behaviour. Bats refused to eat on the first night of captivity (just after capture) and did not defecate during the following day. Only in the evenings, at around 18.00 h did bats produce droppings that were similar in appearance to those described by Thomas (1988) [47]. This behaviour continued throughout the study, though it may have been a consequence of the stress bats experienced during the first day of capture. Nonetheless, retaining food in the gut during the daytime rest phase may be widespread in Old World fruit bats [47]. Such retained seeds would probably be deposited close to the day roosting site, which was used consistently by bats in our study.

The seed shadow map presented here (Fig 7) incorporated spatio-temporal movements of bats and the probability of the deposition of seeds into certain areas. It is not a generic seed dispersal pattern based on travelled distances, but the actual representation of an average bats’ day-to-day behaviour, showing stochastic seed dispersal events. It shows that bats disperse seeds over a vast area and away from the feeding sites. Although most seeds will be deposited below or in close proximity to the feeding trees (Fig 7), in many cases they will be dispersed while commuting between feeding sites. The average distance over which a bat would disperse seeds may be three to five km (Fig 4) and the maximum travel distance recorded over the time when seeds are most likely to be defecated is nearly 20 km. However, this only considers journeys up to three hours after eating figs. Taking into account that bats can retain small numbers of seeds for a day, some seeds may potentially be dispersed over much greater distances and in a wide range of directions.

The spatial scale of dispersal by *P*. *rufus* is much greater than those recorded in lemur species found in Madagascar. For example the highly frugivorous *Eulemur fulvus collaris* moves on average only between 1500 and 3500 m during a day [48]. Although these lemurs contribute to long distance dispersal (LDD) of large and small seeds, their movements are limited to the forest fragments in which they live. Small nocturnal lemur species (*Microcebus* spp.) have limited home ranges, typically from one to four hectares [49], and thus their dispersal abilities are more restricted.

The seed dispersal distances of birds have been studied in more detail, vary among species and habitats and provide a useful yardstick against which to compare our data. Hornbills *Ceratogymna atrata* and *C*. *cylindricus* can disperse seeds as far as 6,919 and 3,558 m respectively [50]. On the other hand, small passerines (<110 g) in Spain disperse most of the seeds to less than 51 m and into covered microhabitats while medium-size birds (110–500 g) can show longer dispersal distances (>110 m) [51]. African turacos (*Corythaeola cristata*, *Musophaga johnstoni* and *Tauraco schuettii*) disperse seeds up to 304 m [52], bulbuls *Hypsipetes amaurotis* up to 300 m [53], and toucans (*Ramphastos* spp.) >100 m [54]. Recently, seed dispersal by *Eidolon helvum* was recorded at occurring over >70 km [12] demonstrating the ability of fruit bats to act as efficient long distance seed dispersers compared with many bird species.

In the majority of ecosystems, birds are the main vertebrate seed dispersers [55]. However, it has been suggested that bats, rather than birds, have a tendency to disperse seeds into clearings [56,57]. This is because birds are more likely to deposit seeds when perching on trees while bats defecate more during flight [e.g. 58,59]. Bats’ ability to defecate frequently when flying has been observed in *Cynopterus* spp., *Rousettus amplexicaudatus* and *Pteropus vampyrus* in Indonesia, and *P*. *poliocephalus* in Australia [43]. It is therefore highly likely that *P*. *rufus* exhibits the same behaviour. In this study. *P*. *rufus* spent nearly 30% of its nocturnal time budget flying and therefore is very likely to disperse many seeds during flight.

The foraging behaviour and flight speed of *P*. *rufus* facilitates long distance dispersal and ensures that seeds are deposited in cleared areas, far from forest boundaries as well as within isolated forest fragments. During flight, the bats can deposit seeds over a large area and far from the parental trees. The seed shadow map represents results from only 11 individuals out of around 600 in the colony (at the time of the study). It is therefore a case-specific documentation of seed dispersal by *P*. *rufus* at Berenty Reserve as the animal’s foraging behaviour might be influenced by the availability of other food types, time of the year, weather, habitat and reproductive status [5]. At some times of the year, the *P*. *rufus* roost at Berenty Reserve may contain over 2,000 individuals [60]. It is therefore clear that within a few months bats will create extensive seed shadows over their colony home range, as well as on their migratory pathways. Additionally, the seeds defecated by bats show increased germination and a better establishment rate to those extracted from fresh fruits. Therefore, this study provides strong evidence to support the hypothesis that *P*. *rufus* is an efficient long distance seed disperser vital for regeneration and maintenance of highly fragmented forest in Madagascar.

In conclusion, the combination of movement data and measurements of GRTs provides a valuable approach for studying where and when animals disperse seeds in the landscape. Similar approaches have been used mainly, but not exclusively on birds [e.g. 9–13; 61]. Our study confirms that flying foxes are important long-distance seed dispersers in degraded landscapes, and provides strong evidence for promoting their conservation, especially at a time when island flying fox populations globally are under threat and in need of protection [62]. Indeed, *P*. *rufus* is listed as ‘Vulnerable’ in the IUCN Red List (iucnredlist.org) and our understanding of the valuable ecosystem services it provides can be used to assist its conservation.

## Supporting information

S1 File Methods for calculation of seed shadows using Gaussian probability density functions. (PDF)
