# Supplementary material for: Flying foxes create extensive seed shadows and enhance germination success of pioneer plant species in deforested Madagascan landscapes
Source: PLoS One. 2017 Sep 6;12(9):e0184023. doi: 10.1371/journal.pone.0184023 (PMC5587229; doi:10.1371/journal.pone.0184023)
Supplement: S1 File — (PDF) [file pone.0184023.s001.pdf]

Flying foxes create extensive seed shadows and enhance germination success of pioneer plant species in deforested Madagascan landscapes

Ryszard Oleksy, Luca Giuggioli, Thomas J. McKetterick, Paul A. Racey & Gareth Jones

S1 Supporting Information

Methods for calculation of seed shadows using Gaussian probability density functions

Feeding times

In addition to space-time values, each recorded trajectory contains information indicating whether the animal was assumed to be feeding at each recorded time step. During a single trajectory there can be several feeding events and the first analytic step is to extract the number of feeding events and time interval during which they occurred. A feeding event can be defined by the time interval $[t_\downarrow, t_\uparrow]$, where $t_\downarrow$ is the beginning and $t_\uparrow$ is the end of such event. The time stamps $t_\downarrow$ and $t_\uparrow$ vary both in number and time of the day for each individual. Feeding events are determined by when bats become stationary as detected by the GPS loggers.

Defaecation probabilities

Four individuals from the GRT experiment provided sufficient data to establish the period over which bats can produce seeded faeces after food ingestion (with no further food ingestion). These data were used to calculate Gaussian probability density functions that predict the likelihood of defecation events occurring after feeding. The probability density functions (or distributions) were used to calculate seed shadows.

The recorded data describe when the flying foxes defecated for the first, second, third and fourth times after ingesting food. To construct a probability distribution associated with the events of dropping seeds from the raw data in Fig 3 (main text) we generated histograms weighted by the

average number of seeds found in the faeces and we separated the events by considering whether they were the first, second, third, or fourth episode after a feeding event. We term the resulting distributions the defecation probability distributions, which we plot in S1 Fig. Two Gaussian probability density functions, $P_1(t)$ and $P_2(t)$, have been fitted to these data, (see S1 Fig), such that $P_1(t)$ is the probability density of the first defecation event occurring at time t after eating. For simplicity the probability densities of a second, third or fourth defecation event have been grouped together and so that $P_2(t)$ represents the combined probability density of a subsequent (second, third or fourth) defecation event occurring at time t. These fitted distributions are:

$$P_1\,(t) = \mathcal{N}\,(0.50, 0.11) \quad [R_2 = 1.0] \qquad (1)$$

$$P_2\,(t) = \mathcal{N}\,(1.81, 0.66) \quad [R_2 = 0.94] \qquad (2)$$

Here $\mathcal{N}\,(\mu, \sigma)$ represents the normal distribution with mean μ and standard deviation σ, where μ and σ are expressed in units of hours.

Examples from two sample individual trajectories of the reconstructed probability distributions of defecation events are shown in S2 Fig and S3 Fig. As both $P_1(t)$ and $P_2(t)$ peaked at their mean times, called t^*_1 and t^*_2 respectively, for each feeding event the probability density of first defecation is maximal at time T_1=$t_{\uparrow}$ + t^*_1, and is also maximal at time T_2=$t_{\uparrow}$ + t^*_2 for any of the other defecation events).

Finding the seeds requires knowledge of the location of the animal at these times. As the trajectory data provides the location of the animal at the end of feeding, time $t_{\uparrow}$, a linear interpolation allowed us to estimate the position of the animal at times T_1 and T_2.

Diffusive displacement

The knowledge of the peak probability distributions $P_1(T_1)$ and $P_2(T_2)$ for the first and subsequent defecation events need to be supplemented with some assumption about the way in which animals explore space.

By assuming that individuals roam randomly over the terrain, it is possible to extract a diffusion coefficient from the data, which can then be used to calculate the average area occupied by the animals in a given time interval.

To calculate the diffusion coefficient, D, we use the relationship between the mean squared displacement (MSD) and time, i.e. $MSD = 4Dt$, for two-dimensional Brownian walkers. For each trajectory the squared displacement between all recorded time steps is calculated and then grouped (binned) according to the time interval. The mean value is obtained by fitting a straight line with zero intercept (S4 Fig). Additional conditions have to be applied to improve the fit so we have ignored the smallest displacements (< 50 m, corresponding to stationary feeding) and the longest time-steps (> 5 hours). Taking the mean across all trajectories to get the best insight into average movement patterns of the flying foxes, the resulting magnitude of the diffusion coefficient is $D = 4.32 \pm 0.4$ ($km^2\ hour^{-1}$). This figure indicates the reasonable outcome that flying foxes could cover on average an area up to 5 km^2 in 1 hour.

Combining space and time

By choosing an overall defecation probability, $\tilde{P}_1$ and $\tilde{P}_2$, we determine the times T_1^- and T_1^+, T_2^- and T_2^+ centred around their respective mean such that $\int_{T_1^-}^{T_1^+} dt\, P_1(t) = \tilde{P}_1$ and $\int_{T_2^-}^{T_2^+} dt\, P_2(t) = \tilde{P}_2$.

From the interpolated spatial position at time T_1 and T_2 and the time intervals $\Delta T_1 = T_1^+ - T_1^-$ and $\Delta T_2 = T_2^+ - T_2^-$ the average area an animal has explored is a disc of radius $R_{1,2}$ given by $\pi R^2_{1,2} = 4D\, \Delta T_{1,2}$. We are thus able to estimate a probability equal to $\tilde{P}_{1,2}$ that an animal defecated (and dropped seeds) within a circle of radius, $R_{1,2}$, centred at the interpolated location at time $T_{1,2}$.

In Fig 5 and 6 of the main text we show the actual animal trajectories associated, respectively, with S2 and S3 Figs with circles of radius $R_{1,2}$ around the location with the maximum probability distribution of defecation occurring at time $T_{1,2}$ after each feeding event. The circles for the first and subsequent defecation events are plotted, respectively, in blue and green. Although the natural choice for $\tilde{P}_1$ and $\tilde{P}_2$ would be to select a value equal for both, the great disparity in the

width of the distribution $P_1(t)$ and $P_2(t)$ forced us to have $\tilde{P}_1 > \tilde{P}_2$. The actual choice, that is $\tilde{P}_1$=0.9 and $\tilde{P}_2$=0.3, was ultimately arbitrary, but it was dictated by the clarity of the display in S2 Fig and Fig 3 in the main text. With these choices of $\tilde{P}_1$ and $\tilde{P}_2$ we obtained the values ΔT_1=32 min and ΔT_2=76 min. The blue and green circles thus represent the average area that a flying fox would cover over 32 and 76 minutes, respectively.

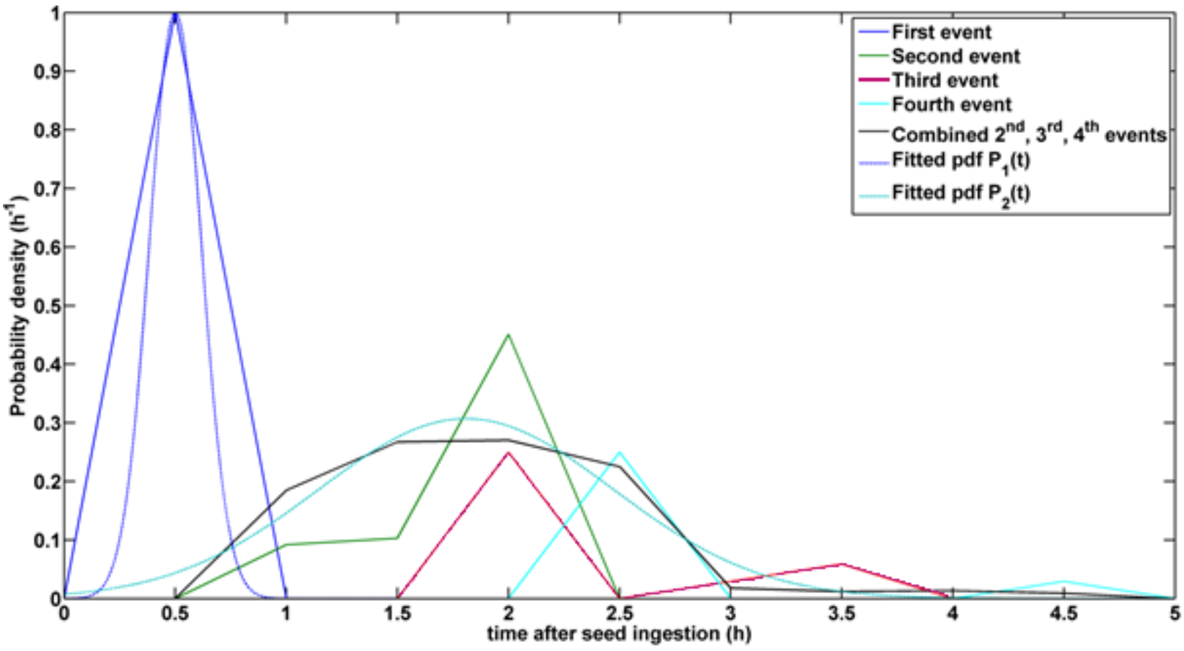

S1 Figure. The recorded data for first, second, third and fourth defecation events plotted as a function of time after food ingestion. Two fitted Gaussian curves are also plotted corresponding to the 'first event', and the 'subsequent event' defecation probability density functions (pdfs).

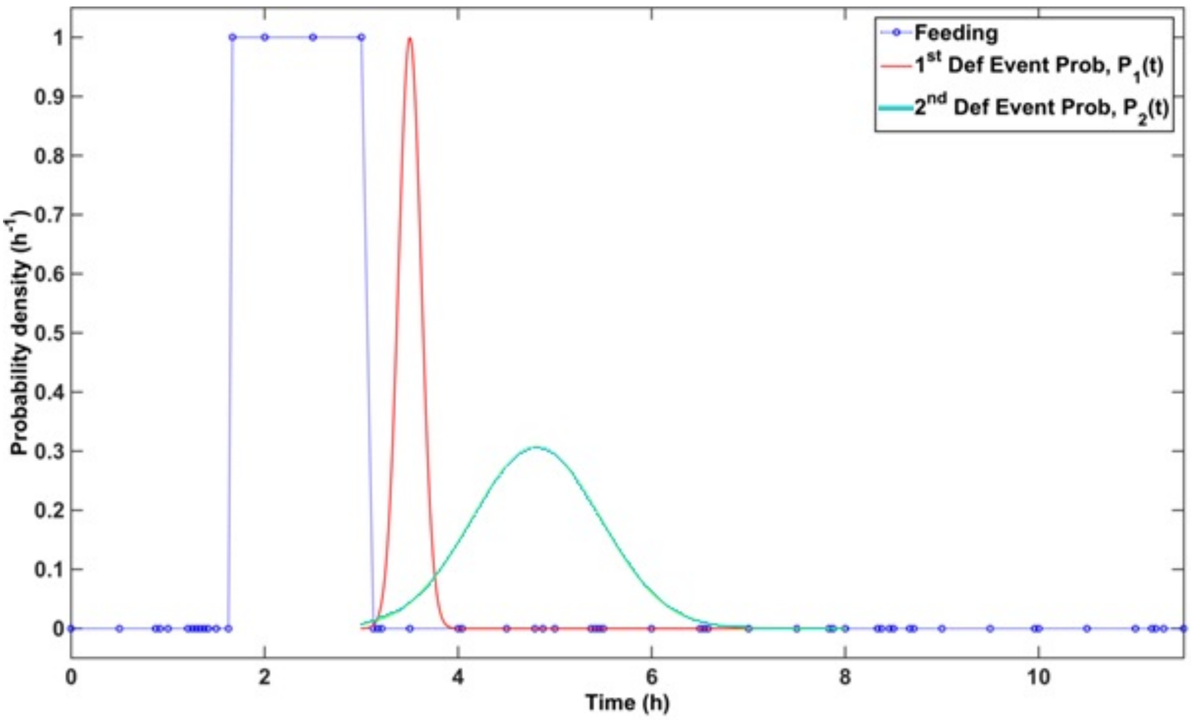

S2 Figure Feeding and defecation event probabilities for the first sample trajectory with only one feeding event.

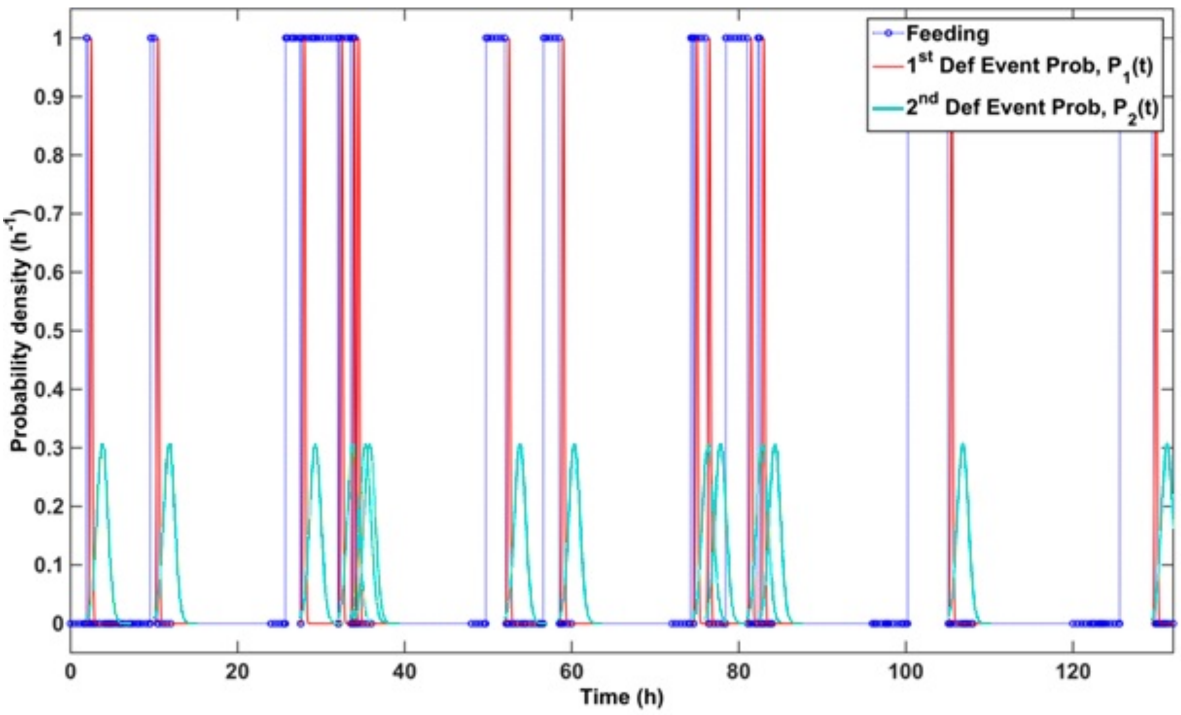

S3 Figure. Feeding and defacation event probabilities for the second sample trajectory with several feeding events.

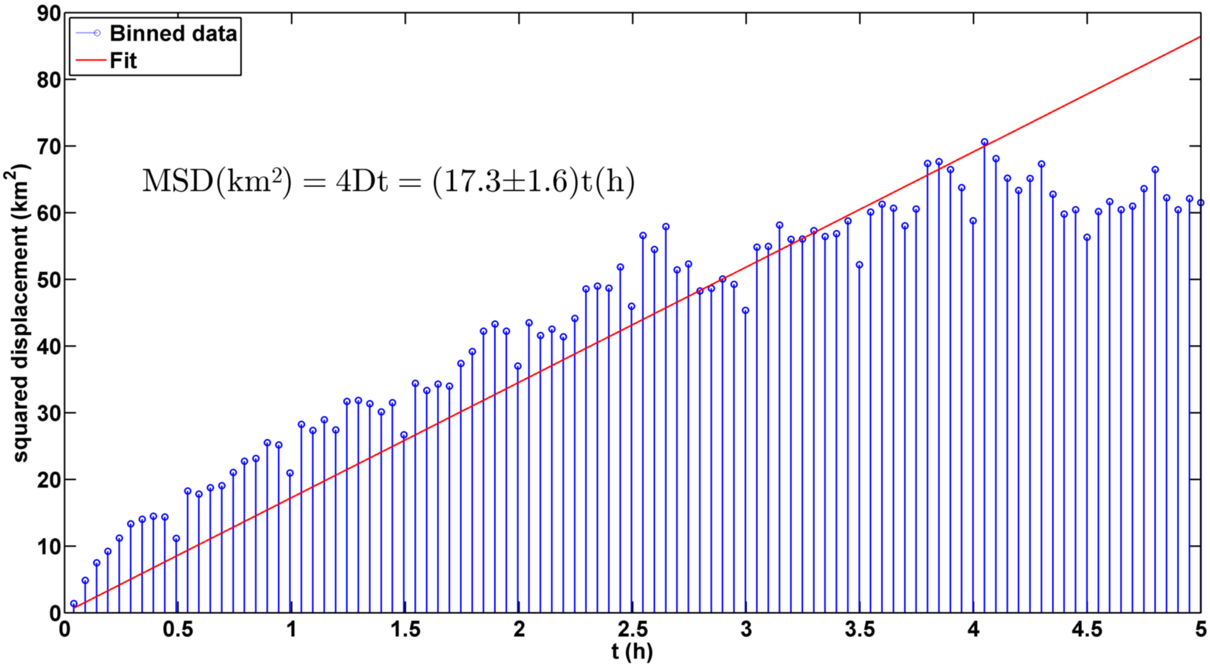

S4 Figure. Squared displacement as a function of the time step interval. Each data point has been constructed by binning the values from different individuals. The red curve is the straight line fit through the origin to determine the diffusion coefficient.
